# Faith, ethics and Section 63 of the Mental Health Act 1983

**DOI:** 10.1192/pb.bp.114.050492

**Published:** 2016-04

**Authors:** Martin Curtice, Louisa James

**Affiliations:** 1 Worcestershire Health and Care NHS Trust

## Abstract

Section 63 of the Mental Health Act 1983 states that an approved clinician can provide medical treatment irrespective of whether or not a detained patient has capacity to refuse such treatment. Case law has established that a range of acts ancillary to the core mental disorder treatment are allowed under Section 63. This article analyses a unique court judgment involving a detained Jehovah's Witness patient who had made an advance decision refusing blood transfusions but who self-inflicted lacerations resulting in blood loss. Core issues within the case involved capacity to consent to treatment and the ethics of treating or not treating patients in such cases.

This article analyses the Court of Protection judgment in the case of *Nottinghamshire Healthcare NHS Trust v RC* [2014].^1^ The case involved the potential use of Section 63 on a detained 23-year-old man. It included elements of the Mental Health Act 1983 (MHA), the Mental Capacity Act 2005 (MCA), the European Convention on Human Rights (ECHR), the Suicide Act 1961 and medical ethics. It is another interesting intersection of the MHA and MCA for consent to treatment issues and it is unique in that it is the first case that considers the court authorising treatment not to be given even though it could lawfully be given under Section 63. This article elucidates the key legal issues contained within the judgment.

## Mental Health Act Section 63

Section 63 (‘Treatment not requiring consent’) is found within Part IV of the MHA (‘Consent to treatment’) and provides that:

‘The consent of a patient shall not be required for any medical treatment given to him for the mental disorder from which he is suffering, not being a form of treatment to which Section 57, 58 or 58A above applies, if the treatment is given by or under the direction of the approved clinician in charge of the treatment.’

The interpretation section, Section 145(4), provides that:

‘Any reference in this Act to medical treatment, in relation to mental disorder, shall be construed as a reference to medical treatment the purpose of which is to alleviate, or prevent a worsening of, the disorder or one or more of its symptoms or manifestations.’

Section 63 provides the authority to give appropriate treatment to detained patients with or without their consent which is not covered by Sections 57 or 58 or 58A. Under Section 58, a 3-month rule specifically applies to medication for mental disorder for detained patients covering the first 3 calendar months commencing from the first date (not necessarily the date on which they were detained) they are administered such treatment as a detained patient; after 3 months such treatment needs to be authorised by a second opinion approved doctor (SOAD). However, as described later, there is a range of medical acts ancillary to the core mental disorder that are allowed under Section 63, but these are not confined to the 3-month rule and can be provided at any time to detained patients. This is the way capacitous and refusing patients can continue to be treated under the MHA.

## The Facts Of The Case

The case involved RC, a 23-year-old man whose parents were practising Jehovah's Witnesses but who was not baptised into the faith. He was taken into care at the age of 4 but was not brought up as a Jehovah's Witness. He had a long history of repeated self-harming behaviours including self-strangulation, burning himself, head-butting, self-laceration and bloodletting. He had been diagnosed as having an antisocial personality disorder and an emotionally unstable personality disorder. He was prescribed warfarin for thrombosis he had previously had.

In 2006, at the age of 15, RC had his first admission to a psychiatric hospital. In 2012 he was convicted of a serious sexual assault on a staff member while a detained patient and sentenced to 5 years' imprisonment.^2^ While in prison in August 2013 he embraced the religion of the Jehovah's Witnesses but still had not been baptised into the faith. It is well known that Jehovah's Witnesses will refuse blood transfusions as part of medical treatment, this being a core belief of the faith and it will be adhered to even if death ensues.

On 1 February 2014 RC badly slashed his brachial artery in the elbow region while in prison. He made further attempts to open the wound. Despite him refusing a blood transfusion when in hospital, no harm came of this. On 12 March 2014 he was admitted to a secure psychiatric hospital under Section 47/49 MHA. Following this he continued to attempt to reopen the wound on a number of occasions. He was placed in a ‘severe mechanical’ restraint belt to prevent this behaviour between 13 and 18 March 2014 (this effectively pinned his arms to the sides of his body to prevent him using his hands to self-harm). The restraint belt needed to be used twice more including the day before his case urgently came before the Court of Protection.

On 4 April 2014 RC signed an advance decision under Sections 24 (‘Advance decisions to refuse treatment: general’) and 26 (‘Effect of advance decisions’) MCA. This was properly witnessed and provided that no transfusions of blood or primary blood components should be administered to him in any circumstances, even if his life was at risk. The judge affirmed that provided RC was of capacity when he made it, ‘this decision has the same effect as if the decision was made when the proposed treatment is to be carried out’.

### The legal proceedings

On 9 April 2014 legal proceedings were commenced. The NHS trust sought:

1. Declarations by the Court of Protection as to RC's capacity in accordance with the MCA:
  - to refuse blood products, and
  - to self-lacerate.
2. A declaration by the Court of Protection that the advance decision of 4 April 2014 made under the MCA was valid and operative should a situation arise where RC needed a blood transfusion but was incapable (for whatever reason) of issuing a decision to refuse one.
3. A declaration that a decision already taken (at least in principle) by the clinician (Dr S) in charge of RC's treatment not to impose a blood transfusion on him, should it be needed, was lawful.

### The legal principles pertaining to the case

The judge elucidated salient principles within this case and started by stating: ‘In principle, every citizen who is of age and of sound mind has the right to harm or (since the Suicide Act 1961) to kill himself’. He further noted a well-established principle from the legal epoch-making case of *St George's Healthcare NHS Trust v S* [1969]^3^ which stated: ‘Even when his or her own life depends on receiving medical treatment, an adult of sound mind is entitled to refuse it’. Furthermore this right applied equally to detained citizens.^4^

Another case was used as a recent example of the lawfulness of withholding blood transfusions from or the administration of blood products to a gravely ill 63-year-old female Jehovah's Witness.^5^ This affirmed there was ‘no obligation on a patient with decision-making capacity to accept life-saving treatment, and doctors are neither entitled nor obliged to give it’. However, the judge acknowledged the ‘right to self-destruction’ could of course have consequences for others, such as friends, family and even wider society. He noted from *Home Secretary v Robb* [1995]^4^ that the ‘consideration of protecting innocent third parties is one that is undoubtedly recognised in this jurisdiction’. On this point the judge commented that if a ‘detained person were to make a formal written request to be given a razor blade with which to harm himself, or a rope with which to hang himself, such a request should obviously be refused both on moral and legal grounds for this reason, as dealing with the aftermath would be a dreadful and traumatic task for the staff’. (He also commented that the provision of a rope would be an offence under the Suicide Act 1961 Section 2(1) – complicity in another's suicide).

The judge observed there were three circumstances where adults may have treatment or other measures imposed upon them without their consent.

1. Adults lacking capacity who pursue a self-destructive course may have treatment forced upon them in their best interests pursuant to the terms of the MCA.
2. Adults who have capacity but who can be categorised as ‘vulnerable’ and who as a consequence of their vulnerability have been robbed of the ability to give a true consent to a certain course of action may also have treatment or other measures imposed on them in their best interests, pursuant to the inherent jurisdiction of the High Court.
3. Under the MHA a detained patient may have treatment imposed on him or her pursuant to Section 63.

In terms of Section 63, the judge noted that a positive decision to impose non-consensual medical treatment under Section 63 was susceptible to judicial review^6^ and that rights under the ECHR were ‘in play’. Pertinent to this case, the judge noted that a decision made by the approved clinician in charge of treatment of a detained MHA patient not to impose any treatment on him or her was not accompanied by any procedure for judicial scrutiny of it. This he found surprising given Article 2 ECHR (the right to life), the ‘most fundamental of the convention rights’, was likely to be engaged in this case. Importantly, he noted that ‘countless authorities’ had emphasised the ‘imperative duty to give effect to this right where detained persons are concerned’. He thought it ‘truly bizarre’ if a full review were held where a positive decision to treat was made under Section 63, but not if there was a negative one not to treat, especially where ‘the negative decision may have far more momentous consequences (i.e. death) than a positive one’.

### The scope of Section 63

Previous jurisprudence provided guidance as to the use of Section 63. The judge noted that cases have ‘drawn a distinction between a condition which is, on the one hand, a consequence of the disorder, and, on other hand, a condition which is a symptom or manifestation of it’. The former was not within Section 63 but the latter was, although such a distinction was ‘intellectually challenging’. It was noted that Section 145(4) had been given a wide interpretation, albeit this was not always consistent and five examples of this were given (Box 1). The core Section 63 issue in this case was whether administration of a blood transfusion following an episode of self-laceration was a treatment of a symptom or manifestation of RC's mental disorder or treatment of a consequence of it.

### The clinical evidence

The Court considered evidence from the treating clinician, Dr S and from Dr Latham, an independent forensic psychiatrist. They were almost unanimous in their views (Box 2). Where they disagreed was as to whether the administration of a blood transfusion amounted to treatment which prevented the worsening of a symptom or manifestation of RC's mental disorder. Dr S opined it ‘plainly was’. Dr Latham disagreed. His reasoning was that ‘ … any treatment with a blood transfusion is not, in my opinion a treatment for mental disorder, nor is it treatment for a symptom of that mental disorder. It is a treatment for a physical consequence of a symptom of the mental disorder; hypovolaemic shock or life-threatening anaemia. This consequence is not wholly as a result of the self-harm but contributed to by his treatment with warfarin. The treatment with warfarin is unrelated to his mental disorder’.

**Box 1** Five examples of previous Section 63 cases

- *Ex parte Brady* [2000]^12^ – this case involved the infamous ‘Moors murderer’ Ian Brady. He went on hunger strike in essence as a protest at being moved wards. The Court found that force-feeding him was lawful within Section 63 and that the hunger strike was a manifestation or symptom of his personality disorder. (For an in-depth review of this case see Curtice.^13^)
- *B v Croydon Health Authority* [1995]^14^ – in this case the Court found it lawful to force-feed a patient who would otherwise die from self-starvation as a result of her borderline personality order. The judgment opined that Section 145(1) MHA gave a wide definition of medical treatment under the MHA which included nursing, care, habilitation and rehabilitation under medical supervision. Hence a range of acts ancillary to the core treatment fell within this meaning and could be considered under Section 63. The Court concluded the term ‘medical treatment … for mental disorder’ in Section 63 included such ancillary acts.
- *A NHS Trust v Dr A* [2014]^15^ – this contrasting case involved a hunger strike by a detained Iranian doctor protesting about the impoundment of his passport. In this case it was found that force-feeding would be treatment for a physical disorder arising from a decision not to eat and drink. The physical condition was considered a consequence of the mental disorder (delusional disorder) but not obviously a symptom or manifestation of it.
- *Tameside and Glossop Acute Services v CH* [1996]^16^ – in this case it was found that section 63 could be used to restrain a patient with paranoid schizophrenia to enforce a Caesarean section upon her when obstetric complications arose.
- *St George's Healthcare NHS Trust v S* [1998]^7^ – a pregnant mother who rejected medical advice as to treatment necessary to protect her and her unborn child was unlawfully admitted and detained under Section 2 MHA and unlawfully forced to have a Caesarean section by the order of a court.

The judge in this case stated that were he ‘confined to the literal words of Section 63 and 145(4)’ he thought he would agree with Dr Latham and RC's counsel. However, previous jurisprudence had supplied a definition which was ‘some distance from the meaning of the literal words’ – the judgment in *St George's Healthcare NHS Trust v S* [1998]^7^ stated: ‘Section 63 of the Act may apply to the treatment of any condition which is integral to the mental disorder’.

### Assessment of capacity under the MCA

The judge considered the capacity of RC to make a decision to refuse a blood transfusion and in doing so applied the MCA. He outlined the fundamental principle under Section 1(2) MCA that capacity is to be assumed unless it is established, on the balance of probabilities, to be lacking. He also applied Section 2 MCA (people who lack capacity) and Section 3 MCA (inability to make decisions) reiterating that a person will lack capacity to make a decision if, by reason of mental disorder, they are unable to understand, or retain, or use or weigh up the information relevant to that decision, or to communicate their decision. The judge considered the only real question in terms of the MCA related to whether RC was able to weigh information in the balance but acknowledged: ‘This aspect of the test of capacity must be applied very cautiously and carefully when religious beliefs are in play’. He placed ‘little emphasis’ on the fact that a central tenet of RC's religious faith prevented him from weighing the advantages of a blood transfusion should his medical circumstances indicate one was necessary. This was because ‘it would be an extreme example of the application of the law of unintended consequences were an iron tenet of an accepted religion to give rise to questions of capacity under the MCA’. He concluded that RC had full capacity to refuse the administration of blood products. Although he did not feel a formal declaration was needed as to the capacity of RC to harm himself in light of the agreed expert evidence as above, he did agree that RC sometimes did, and sometimes did not, have the capacity to inflict lacerations on himself.

**Box 2** Areas of agreement between the two clinicians giving evidence

Both clinicians agreed that:

- RC suffered from a mental illness, namely antisocial and emotionally unstable personality disorders. This was a disturbance of the functioning of the mind, which is one of the classic definitions of mental disorder.
- He had full capacity to refuse blood products and this refusal derived almost exclusively from his religious faith. He had full capacity to enter into the advance decision on 4 April 2014. His decision to adopt the religion of the Jehovah's Witnesses was made with full capacity.
- So far as RC's capacity to harm himself was concerned, on occasions he did so with full capacity. On other occasions, particularly at times of severe emotional distress, it was likely that he did so without the capacity to choose to self-harm.
- RC harmed himself with the intention of distracting himself from distressing thoughts and feelings. He did so without really thinking about the consequences and dangers. However, his view was that it was his body and therefore his choice to damage it.

### The advance decision

The advance decision made by RC on 4 April 2014 was assessed. It was noted that under Section 26(4)(a) and (b) MCA the Court of Protection may make a declaration as to whether such an advance decision is valid and is applicable to a treatment. Sections 24 and 25 MCA (‘Validity and applicability of advance decisions’) contain a number of formal requirements for validity. In relation to a capacitous advance decision to refuse life-saving treatment, in this case the judge considered the only question related to the witnessing requirements in that the signature was made or acknowledged in the presence of a witness (Section 25(6)(c)) and that the witness had signed it in the person's presence (Section 25(6)(d)). He concluded that all formal requirements relating to the advance decision were satisfied. Hence RC was assessed as having full capacity when he executed the advance decision – so should RC ever be in a position where for whatever reason he lacked capacity, but a blood transfusion was clinically indicated, then the advance decision would be operative.

### The ethical basis for not invoking Section 63

The last legal declaration sought in this case related to the decision of Dr S not to invoke the powers under Section 63. In her evidence she stated that she had ‘ … some ethical difficulty in using the MHA to override a capacitous patient's wishes based on religious wishes and I would not choose to use my MHA powers to override his advanced (sic!) decision’. The judge concluded this was completely correct. Furthermore he emphasised that ‘ … it would be an abuse of power in such circumstances even to think about imposing a blood transfusion on RC having regard to my findings that he presently has capacity to refuse blood products and, were such capacity to disappear for any reason, the advance decision would be operative. To impose a blood transfusion would be a denial of a most basic freedom’. It was concluded that Dr S's decision was lawful and it was lawful for those responsible for the medical care of RC to withhold all and any treatment which involved transfusion into him of blood or primary blood components (red cells, white cells, plasma or platelets) in spite of the existence of powers under Section 63.

## Discussion

Despite being one of the more widely used provisions of the MHA, Section 63 does not require specific formal paperwork for its implementation, and there has been a scarcity of publications on it. This probably reflects the fact that relatively few such cases have actually reached the courts. Another unreported court case adding to the range of ancillary acts allowed under Section 63 described earlier found it lawful to treat, with reasonable restraint if necessary, a 17-year-old woman with diabetes who was refusing her insulin therapy.^8^ The court accepted that the refusal of insulin treatment was part of self-harming behaviour which was a symptom of her mental disorder (a personality disorder). In terms of court cases involving the Jehovah's Witness faith, a recent case involving a very young child of devout Jehovah's Witness parents who suffered severe burns to his body in an accident concluded that despite opposition from the parents, it was entirely in the child's best interests to receive skin grafts and if necessary blood transfusions if they were clinically needed.^9^

There were two areas from the RC judgment which have particular resonance for clinical practice. First, the judge clearly concluded it could not be disputed that the act of self-harming – the slashing open of the brachial artery – was a symptom or manifestation of the underlying personality disorder. It therefore followed that to treat the wound in any way was to treat the manifestation or symptom of the underlying disorder and ‘So, indisputably, to suture the wound would be squarely within Section 63’ as was the administration of a course of antibiotics to prevent infection. This line of thinking was further emphasised in his view that to treat a low haemoglobin as a consequence of bleeding from a wound by a blood transfusion was ‘just as much a treatment of a symptom or manifestation of the disorder as is to stitch up the wound or to administer antibiotics’.

Second, the judge advised that where an approved clinician makes a decision not to impose treatment under Section 63, and where the consequences of such a decision could prove life-threatening, then an involved NHS trust was advised to apply to the High Court for a declaration and full review of the original decision. This was especially important to ensure a patient fully understood the consequences of opting out of their Article 2 right to life (this contrasts with a case where the Supreme Court found a breach of Article 2 where an informal patient died by suicide while on leave from hospital and it was found that inadequate risk assessment and management contributed to the death).^10^ He further advocated, as happened in this case, obtaining a second opinion of the approved clinician's decision in such cases. Commenting specifically on this, Ruck Keene & Burnell^11^ felt this fell short of a finding that trusts should have to apply, but that it was a very clear judicial steer. They also produced a series of questions, based on case-law principles, that should be considered before taking a decision to forcibly treat someone under Section 63 MHA (Box 3). Ruck Keene & Burnell also advise that any treatment given under Section 63 must be justified on the basis of its necessity and proportionality to the seriousness of the risk to the patient (although not specifically mentioned, this is consistent with Article 8 ECHR – the right to respect for private and family life).

The judge in this case eloquently, albeit possibly a little stereotypically at one point, commented that:

‘At first blush Section 63 strikes one as an illiberal provision, given that it applies to all detained mentally ill patients who may well not lack capacity (as here). However, it can be well justified when one reflects that the treatment in question may be needed not merely for the protection of the patient but also for the prevention of harm to others, given the violent eruptions to which mental illness can give rise.’

He also elucidated the clear upholding of the principle that a person with capacity should be able to refuse medical treatment even if there is a legal framework which could be used to impose it against their will. Given that the 2007 amendments to the MHA made a significant concession to those detained patients having capacity to now be able to refuse electroconvulsive therapy (Section 58A), it is possible that future MHA amendments may similarly amend Section 63 to allow refusal of medical treatment by capacitous detained patients.

**Box 3** Questions to be considered before forcibly treating someone under Section 63^a^

- If the patient is not detained, do they meet the criteria for detention? We suggest that very considerable caution should be exercised before detaining a patient simply for purposes of using Section 63.
- Does the proposed treatment clearly fall within the scope of Section 63, and the definition of ‘medical treatment’ within Section 145 MHA?
- Is there a clear connection between the mental disorder and the treatment you are giving? What is that connection?
- Will the treatment of the physical issue amount to treatment of the mental disorder? And if so, how?
- Have you checked whether the patient has made an advance decision? If so, what does it cover?

To this list could be added:

- Is the proposed medical treatment under Section 63 aimed at treating a consequence, or a symptom or manifestation, of the mental disorder?
- Is it clear having applied the test of capacity to consent to medical treatment under the MHA (being able to understand the nature, purpose and likely effects of treatment) that the patient is either capacitous or incapacitous to consent to medical treatment?
- Can the medical treatment being proposed under Section 63 be justified on the basis of its necessity and proportionality to the seriousness of the risk to the patient?

a. *Rabone & Anor v Pennine Care NHS Foundation Trust* [2012].^10^
